# Uptake of cervical cancer screening and its predictors among women of reproductive age in Gomma district, South West Ethiopia: a community-based cross-sectional study

**DOI:** 10.1186/s13027-022-00455-x

**Published:** 2022-08-08

**Authors:** Abraham Tamirat Gizaw, Ziad El-Khatib, Wadu Wolancho, Demuma Amdissa, Shemsedin Bamboro, Minyahil Tadesse Boltena, Seth Christopher Yaw Appiah, Benedict Oppong Asamoah, Yitbarek Wasihun, Kasahun Girma Tareke

**Affiliations:** 1 Department of Health, Behavior, and Society, Faculty of Public Health, Institute of Health, Jimma University, Jimma, Ethiopia; 2 School of Nursing Institute of Health, Jimma University, Jimma, Ethiopia; 3 Armauer Hansen Research Institute, Ministry of Health, Jimma, Ethiopia; 4 Department of Global Public Health, Karolinska Institutet, Stockholm, Sweden; 5 World Health Programme, Université du Québec en Abitibi-Témiscamingue (UQAT), Québec, Canada; 6 Department of Sociology and Social Work, Kwame Nkrumah University of Science and Technology, Kumasi, Ghana; 7 Department of Clinical Sciences, Social Medicine and Global Health, Lund University, Lund, Sweden

**Keywords:** Cervical cancer, Factors, Screening uptake, Ethiopia

## Abstract

**Background:**

Cervical cancer is a public health challenge despite the available free screening service in Ethiopia. Early screening for cervical cancer significantly improves the chances of successful treatment of pre-cancers and cancers among women of reproductive age. Therefore, this study aimed to assess the uptake of screening and identify the factors among women of reproductive age.

**Methods:**

A community-based cross-sectional study was conducted in Gomma Woreda, Jimma Zone, Ethiopia, from 1st to the 30th of August, 2019. The total sample size was 422. A systematic random sampling technique was employed. Data were collected using a structured questionnaire, entered in epidata, and exported and analyzed using SPSS version 20.0 software packages. Descriptive, bivariate and multivariable logistic regression analyses with 95% CI for odds ratio (OR) were performed to declare a significant predictors.

**Result:**

A total of 382 study participants were involved with a response rate of 90.5%. The mean age of the study participants was 26.45 ± 4.76 SD. One hundred forty-eight (38.7%) of participants had been screened for CC. Marital status (AOR = 10.74, 95%, CI = 5.02–22.96), residence (AOR = 4.45, 95%, CI = 2.85–6.96), educational status (AOR = 1.95, 95% CI = 1.12–3.49), government employee (AOR = 2.61, 95%, CI = 1.33–5.15), birth experience (AOR = 8.92, 95% CI = 4.28–19.19), giving birth at health center and government hospitals (AOR = 10.31, 95% CI = 4.99–21.62; AOR = 5.54, 95% CI = 2.25–13.61); distance from health facility (AOR = 4.41, 95% CI = 2.53–9.41), health workers encouragement (AOR = 3.23, 95% CI = 1.57–6.63), awareness on cervical cancer (AOR = 0.37, 95% CI = 0.19–0.72), awareness about CC screening (AOR = 4.52, 95%, CI = 2.71–7.55) and number of health facility visit per year (AOR = 3.63, 95%, CI = 1.86–6.93) were the predictors for the uptake of cervical cancer screening.

**Conclusion:**

The uptake of cervical cancer screening was low. Marital status, residence, occupation, perceived distance from screening health facility, health workers encouragement, number of health facility visits, birth experience, place of birth, and knowledge about cervical cancer screening were the predictors. There is a need to conduct further studies on continuous social and behavioral change communication.

## Background

Cervical cancer is the fourth most common cancer among women worldwide. Globally, there are approximately 570, 000 morbidities and 311, 000 mortalities from cervical cancer in 2018. The estimated age-standardized incidence of cervical cancer was 13.1 per 100, 000 women and varied widely among countries with rates ranging from below 2 to 75 per 100, 000 women [1, 2]. About 83% of new cervical cancer cases and 85% of related deaths occur in low- and middle-income countries, affecting poor, vulnerable, and disenfranchised women at the prime of life. Cervical cancer is the leading cause of cancer-related mortalities among women’s in eastern, western, middle, and southern Africa. Globally, the average age at diagnosis of cervical cancer was 53 years [3, 4].

Similarly, about 80% cases in sub-Saharan Africa detected at a late stage, when a need to involve multiple treatments, including surgery, radiotherapy and chemotherapy happen; or when treatment is likely lacking/limited, ineffective, too expensive or inaccessible for many women in low-resource countries, or when it is associated with a markedly diminished chance of prognosis success after treatment [5, 6] Also, in Ethiopia, an estimated number of 6300 new cases and about 4,884 related mortalities occur each year [7, 8]. For example, there were about 6294 new cervical cancer cases reported in 2018. It is the second leading cause of and common female cancer in Ethiopia [9].

Nevertheless, it is one of the most preventable and curable forms of cancer, as long as it is detected early and managed effectively. However, detection of cervical cancer occurs late [4–7]. The World Health Organization (WHO) planned to eliminate cervical cancer among women of all countries by 2030, through the provision of full human papillomavirus (HPV) vaccine to 90% of girls at the age of 15 years and above, conducting screening for 70% reproductive age group women, delivering treatment and care for 90% identified women [1]. The Ethiopian cervical cancer prevention and control guideline by employing interventions focusing on the three stages of disease prevention (primary prevention, secondary prevention, and tertiary care management of invasive cervical cancer including surgery, radiation therapy, and chemotherapy as well as palliative care).

Health facilities are the frontline responsible bodies for designing appropriate communication and advocacy strategies to increase health services that promote, prevent and cure cervical cancer cases. Behavioral change communication (BCC) intervention is one of the activities conducted by primary health care workers to increase awareness of cervical cancer prevention, influence social norms, and facilitate behavior change among selected individuals or sub-populations to prevent cervical cancer. Community health workers and health development armies play an essential role within the community in promoting the acceptability of cervical cancer prevention services through advocacy and providing information about cervical cancer prevention services, identifying eligible groups, and assisting women in making decisions to attend the health facilities for cervical cancer prevention services and engaging cervical cancer survivors [6]

However, findings from existing evidence indicated that there was low CC screening behavior at different areas, including Ethiopia [10–14]. Therefore, the study was conducted to determine CC screening behavior and its associated factors among reproductive age group women.

## Methods

### Study design, setting and period

A cross-sectional study was conducted in Gomma Woreda, Jimma zone, Oromia regional state, Ethiopia from 1st to the 30th of August, 2019. The 2007 national census reported a total population of the Woreda to be 213,023, of which 108,637 and 104,386 were men and women respectively. About 12,769 or 5.99% of the population were urban dwellers. The majority of the inhabitants were Muslim (83.88%), while 14.68% of the population practiced Ethiopian Orthodox Christianity, and 1.34% were Protestant [15]. The Woreda had 36 rural and 3 urban kebeles (small administrative units) [16].

### Study participants

All reproductive age group (15- 45 years) women were source and study populations. A systematic random sampling technique was employed to select households. If more than one eligible woman was found in the households, a lottery method was applied to select one. All reproductive age group women who were living in the study setting for more than six months were eligible for the study. On the other hand, individuals who were unable to speak or hear; lived in the study area for less than 6 months and critically sick were excluded from the study. Considering the proportion of cervical cancer screening rate (50%), 95% confidence interval, and a 5% margin of error, the required sample size was calculated using a single population proportion formula.

$$n = \frac{{Z2P\left( {1 - P} \right)}}{D2} = \frac{{1.962*0.5\left( {0.5} \right)}}{0.052} = 384$$

Considering a 10% non-response rate, the total sample size was 422.

### Data collection procedure

An interviewer-administered structured questionnaire adapted from different studies were used to collect the data [17–25]. To ensure consistency and accuracy, the questionnaire was prepared in English language from different sources and translated to the local languages (Afan Oromo and Amharic) using linguistic experts in the local settings. After translating the questionnaire back into English Six data collectors (4 clinical nurses and 2 BSc nurses) who have previous experience in data collection and fluency in the languages of the community collected the data. Two public health officers with established competence in research data supervision were recruited.

### Data analysis

The data were entered, cleaned, and checked using Epi data manager version 4.0.2, and exported to SPSS version 21 statistical software package for analysis. Binary and multivariable logistic regression analyses were carried out to identify an association between the predictors and outcome variables. Binary logistic regression analysis was performed to select variables for multivariable logistic regression analysis. Variables with a p-value < 0.25 in the binary logistic regression analysis were taken as candidates for multivariable logistic regression analysis. Finally, multivariable logistic regression analysis was performed to control for the possible confounding effects of the selected variables. Variables with a p-value < 0.05 were recognized as statistically significant associations with women’s service utilization for cervical cancer screening. The odds ratio with 95% CI was used to show the degree of association between the independent and outcome variables. A descriptive analysis using frequencies and proportions were also performed for different variables.

### Data quality control

Before the actual data collection, the questionnaire was pre-tested on similar setting outside the study area. The data collectors and supervisors were trained for three days on principles, ethical considerations, procedures, and details of the questionnaire. The data was checked daily for uniformity and completeness before data entry during the data collection. The principal investigators closely monitored the data collection process.

### Variables

#### Dependent variable

Uptake of cervical cancer screening.

#### Independent variable

1. Socio-demographic characteristics: Age, religion, educational status, occupational status, residence, monthly income, ethnicity, marital status, birth experience, place of birth, parity.
2. Knowledge about cervical cancer, cervical cancer symptoms, risk factors, prevention methods, and treatment options.
3. Husband's support, peer pressure, health workers encouragement, health developmental army encouragement, traditional healers influence, a number of health facility visits per year.

#### Operational definitions

Women of reproductive age were considered to have good knowledge of the uptake of cervical cancer screening if they know half or more of the measure of score level items of the cervical cancer screening (cervical cancer, cervical cancer symptoms, risk factors, prevention methods, and treatment options).

## Results

### Socio-demographic characteristics

A total of 382 women were participated in the study with a response rate of 90.5%. The mean age of the respondents was 26.45 years (16–36 years) with 4.76 standard deviation (SD). The majority (61.8%) of respondents were aged 25 to 34 years old. Two hundred forty-two (63.4%) of respondents were from rural area. A large number of respondents, 267 (69.9%) were Muslims, followed by orthodox 88 (23%) believers. About 145 (38%) of respondents had not attended formal education. Also, about 285 (74.6%) were married. The majority 306 (80.1%) of the respondents were from Oromo ethnic group. About 295 (77.2%) of respondents had birth experience, and 247 (64.7%) of the respondents had one or more health facility visits per year (Table 1).

**Table 1:** Socio-demographic characteristics and obstetrics history of participants in Gomma Woreda, Jimma zone, Oromia regional state, South West Ethiopia, 2019

Variables	Category	Frequency	Percent
Age	15–24 years	126	33
	25–34 years	236	61.8
	≥ 35 years	20	5.2
Marital status	Single	97	25.4
	Married	285	74.6
Educational status	No formal education	145	38
	Primary education	132	34.6
	Secondary and above	105	27.5
Residence	Urban	140	36.6
	Rural	242	63.4
Religion	Orthodox	88	23.0
	Muslim	267	69.9
	Protestant	12	3.1
	Other	15	3.9
Occupation	Housewife	145	38.0
	Merchant	67	17.5
	Government employee	47	12.3
	Farmer	113	29.6
	Other	10	2.6
Ethnicity	Oromo	306	80.1
	Amhara	36	9.4
	Guraghe	29	7.6
	Other	11	2.9
Birth experience	Yes	295	77.2
	No	87	22.8
Parity	0	87	22.8
	1–4 children	214	56.0
	≥ 5 children	81	21.2
Place of birth for the last child	Health center	172	45.0
	Government hospital	43	11.3
	Other*	16	4.2
	Home	68	17.8
Monthly income	≤ 499	88	23.0
	500–1000	138	36.1
	≥ 1000	156	40.8
Number of health institution visits	Once a year or more	247	64.7
	Once every two years	71	18.6
	Ever no visit	64	16.8

*Health post and private clinics

### Knowledge on cervical cancer and its screening

A descriptive analysis result showed that about 283 (74.1) and 247 (64.7%) of respondents had awareness about cervical cancer and its screening. The major sources of information were health workers (12.3%), printed material (7.1%) and radio/television (6.8%). 240 (62.8%) and 105 (27.5%) of respondents knew that cervical cancer has symptoms like pain during sex and foul vaginal discharge respectively. About 240 (62.8%) and 170 (44.5%) of study participants indicated that excessive sex and hereditary/family history were the risk factors for cervical cancer. Ninety-five (24.9%) of the respondents knew that cervical cancer could be cured in its earliest stages (Table 2).

**Table 2:** Women’s knowledge of cervical cancer and cervical cancer in Gomma Woreda, Jimma zone, Oromia regional state, South West Ethiopia, 2019

Variables	Category	N (%)
Awareness about cervical cancer		283 (74.1%)
Knowledge about symptoms cervical cancer	Vaginal bleeding	69 (18.1%)
	Foul vaginal discharge	105 (27.5%)
	Post-coital bleeding	25 (6.5%)
	Pain during sex	240 (62.8%)
	Others	5 (1.3%)
Knowledge on risk factors of cervical cancer	Having multiple sexual partners	97 (25.4%)
	Sex at an early age < 15yrs	52 (13.6%)
	Acquiring HPV virus	123 (32.2%)
	Cigarette smoking	69 (18.1%)
	Long time use of birth control pills	63 (16.5%)
	Early pregnancy (< 15 years)	44 (11.5%)
	Sexually transmitted infection	45 (11.8%)
	Repeated Abortion	44 (11.5%)
	Multiparty	45 (11.8%)
	Excessive sex	240 (62.8%)
	Lack of hygiene	92 (24.1%)
	Heredity/family history	170 (44.5%)
	Other	100 (26.2%)
Knowledge on prevention methods of cervical cancer	Avoid multiple sexual partners	139 (36.4%)
	Avoid sex before < 15 years	274 (71.7%)
	Avoiding cigarette smoking	98 (25.7%)
	Through HPV vaccine	114 (29.8%)
	Avoid pregnancy ≤ 15 years	111 (29.1%)
	Prevent STIs by safe sex	151 (39.5%)
	Others	49 (12.8%)
Knowledge of treatment of cervical cancer	Can be cured in early stages	95 (24.9%)
	Herbal remedies	86 (22.5%)
	Surgery	57 (14.9%)
	Other	28 (7.3%)
Perception on costs of cervical cancer treatment	Affordable	51 (13.4%)
	Moderately expensive	84 (22.0%)
	Very expensive	90 (23.6%)
	Unknown	157 (41.1%)
Awareness about cervical cancer screening	247 (64.7%)	
Source of information	Radio/television	26 (6.8%)
	Printed material	27 (7.1%)
	Health workers	47 (12.3%)
	Family, friends and neighbors	17 (4.5%)
	Leaders	10 (2.6%)
	Teachers/school system	9 (2.4%)
	Others	5 (1.3%)
Knowledge on the recommended frequency of eligible women to screening for premalignant cervical lesion	Once in every year	82 (21.5%)
	Once every three years	86 (22.5%)
	Once every 5 years	73 (19.1%)
	Unknown	141 (36.9%)
Knowledge of eligibility cervical cancer screening	All women of ≥ 25 years	104 (27.2%)
	Commercial sex workers	86 (22.5%)
	Early women only	68 (17.8%)
	Unknown	124 (32.5%)

### Uptake of cervical cancer screening and associated factors

According to this study, 148 (38.7%) of respondents had been screened for cervical cancer. Women’s age, educational status, marital status, occupation, birth experience, place of birth, husband support, distance of participant’s home from health facility that undergone CCa screening, health worker’s encouragement, awareness about cervical cancer screening, number of health facility visit per year, awareness about cervical cancer, and knowledge about the cervical cancer management were identified as candidate variables for multivariable logistic regression analysis. Women’s marital status, residence, occupation, distance, health workers encouragement, a number of health facility visits, birth experience, place of birth, awareness about cervical cancer and cervical cancer screening were identified from multivariable logistic regression analysis as predictors of uptake of cervical cancer screening.

Women living in urban area were 4.45 times more likely to uptake cervical cancer screening service than those who live in rural areas (AOR = 4.45, 95% CI = 2.85–6.96). Women who attended secondary and above education were 1.95 times more likely to utilize cervical cancer screening service delivery than those who had not attended formal education (AOR = 1.95, 95% CI = 1.12–3.49). Married women were 10.74 times more likely to uptake cervical cancer screening than unmarried women (AOR = 10.74, 95% CI = 5.02–22.96). Women employed in governmental organization were 2.61 times more likely to use the cervical cancer screening service than house wife’s (AOR = 2.61, 95% CI = 1.33–5.15). Women who had a birth experience were 8.92 times more likely to accept cervical cancer screening service than who women who have no childbirth (AOR = 8.92, 95% CI = 4.28–19.19).

Place of birth were another significant predictor for cervical cancer screening. Women who gave birth at the health facility ( health center and hospital) were 10.31,and 5.54 times more likely to actively utilize cervical cancer screening service (AOR = 10.31, 95% CI = 4.99–21.62) and (AOR = 5.54, 95% CI = 2.25–13.61), respectively. On the other hand, women who had no perceived distance problem from the screening center to the health facility were 4.4 times more likely to uptake cervical cancer screening service (AOR = 4.41, 95% CI = 2.53–9.41). Women who got health workers encouragement were 3.23 times more likely to receive the cervical cancer screening service (AOR = 3.23, 95% CI = 1.57–6.63).

Study participants who had awareness about cervical cancer and cervical cancer screening services were 0.37 and 4.52 times more likely to obtain cervical cancer screening service (AOR = 4.52, 95% CI = 2.71–7.55; AOR = 0.37, 95% CI = 0.19–0.72), respectively. Women who had a history of visiting health facilities for one or more times annually were 3.63 times more likely to uptake cervical cancer screening service than women who has not visited within a year (AOR = 3.63, 95% CI = 1.86–6.93) (Table 3).

**Table 3:** Factors associated with cervical cancer screening among women’s in Gomma Woreda, Jimma zone, Oromia regional state, Ethiopia, 2019

Variables	Cervical cancer screening		COR (95%CI)	AOR (95%CI)
	Yes	No		
*Women’s age*				
15–24 years	25	101	1	**1**
25–34 years	112	124	3.649 (1.36–9.76)	0.20 (.076–1.54)
≥ 35 years	11	9	4.938 (1.97–12.35)	0.74 (.3- 1.85)
*Residence*				
Urban	85	55	4.39 (1.78–10.84)	4.45 (2.85–6.96)*
Rural	63	179	1	1
*Educational status*				
No formal education	56	89	1	1
Primary education	42	90	0.74 (0.45–1.24)	1.65 (.97–2.82)
Secondary education and above	50	55	1.44 (0.85–2.45)	1.95 (1.12–3.49)*
*Marital status*				
Married	140	145	10.74 (7.29–22.87)	10.74 (5.02–22.96)*
Single	8	89	1	1
*Occupation*				
Housewife	59	86	1	**1**
Merchant	30	43	1.02 (0.60–1.73)	1.76 (0.94–3.28)
Government employee	28	27	1.51 (0.81–2.83)	2.61 (1.33–5.15)*
Farmer	31	78	0.58 (0.29–1.13)	1.73 (.91–2.94)
*Monthly income*				
≤ 499	37	51	1	1
500–1000	69	40	2.38 (1.40–4.04)	1.23 (0.88,1.99)
≥ 1000	105	80	1.81 (1.08–3.01)	1.89(0.22,3.45)
*Birth experience*				
Yes	140	155	8.92 (4.18–19.10)	8.92 (4.28–19.19)*
No	8	79	1	1
*Place of birth*				
Health center	110	62	10.29 (4.90–21.54)	10.31 (4.99–21.62)*
Government hospital	21	22	5.54 (2.25–13.60)	5.54 (2.25–13.61)*
Other*	5	11	2.64 (0.75–9.2)	2.64 (0.75–9.23)
Home	10	58	1	1
*Husband support*				
Yes	56	80	0.57 (0.38–0.86)	0.68 (.42–1.1)
No	84	68	1	1
*Peer pressure*				
Yes	55	117	0.59 (0.39–0.89)	0.75(.4–1.4)
No	93	117	1	1
*Distance from screening center*				
Yes	63	179	1	1
No	85	55	4.43 (2. 51–9.4)	4.41 (2. 53–9.41)*
*Health workers encouragement*				
Yes	76	72	1.1 (0.72–1.67)	3.23 (1.57–6.63)*
No	114	120	1	1
*Awareness about cervical cancer*				
Yes	99	112	2.20 (1.33–3.64)	0.37 (0.19–0.72)*
No	49	122	1	1
*Awareness about cervical cancer screening*				
Yes	117	201	0.62 (0.47–1.02)	4.52 (2.71–7.55)*
No	31	33	1	1
*Knowledge on treatment of cervical cancer*				
Poor	41	66	1	1
Good	107	168	1.02 (0.62–1.69)	0.45 (.19–1.07)
*Number of health facility visit*				
Once a year or more	118	129	3.6 (1.86–6.93)	3.63 (1.86–6.93)*
Once every two years	17	54	1.24 (0.55–2.84)	1.24 (0.55–2.80)
Ever no visit	13	51	1	1

*Significant association at *p* ≤ 0.05

## Discussion

The study found that Women’s marital status, residence, occupation, distance to primary health care facility, health workers encouragement, frequency of health facility visits, birth experience, place of birth, awareness about cervical cancer and cervical cancer screening service were the predictive variables of reproductive age women’s uptake of the cervical cancer screening in the study area.

According to this study, the uptake of cervical cancer screening service was 38.7%. This was higher than the finding of studies conducted in Addis Ababa (25%), Amhara region (5%), Hossana town (14.2%), St. Paul’s Teaching and Referral Hospital (12.2%), southern Ethiopia (27.7%), Wolaita Zone (22.9%), Yirgalem hospital (16.5%), Kenya (25.6%), and Zimbabwe (83.2%) [17–25]. The finding of this study revealed promising practice of the cervical cancer screening service among aged 15 years or more compared to the previous study findings [17–24]. This might be due to SBCC activities and cervical cancer screening campaigns conducted in the study area, which implicates the significance of conducting SBCC intervention and provision of CCa screening campaigns to increase the uptake. In addition, geographical and time difference played key roles on this study as it was conducted in rural and community-based setting unlike the previous studies that were conducted in urban institutional settings.

Women who live in urban area were 4.45 times more likely to receive cervical cancer screening service than study participants who live in rural area (AOR = 4.45, 95%CI = 2.85–6.96). This finding was supported by a study conducted at St. Paul’s Teaching and Referral Hospital [20]. This indicated that majority of health facilities that provide CCa screening were found at urban settings. In addition, urban dwellers might have more information about it through different medias/means. Furthermore, populations found at urban settings might have better educational background than women living in rural setting. This is revealed in the study that women who attended secondary and above education level were 1.95 times more likely to actively uptake cervical cancer screening service than those who had not attended formal education (AOR = 1.95, 95% CI = 1.12–3.49). This result is consistent with the findings of studies conducted at different settings in Ethiopia [18, 21, 22]. These findings imply that there is a need to empower women regardless of their residency through formal education and also conducting social and behavioral change communication interventions. In addition, there is a need to expand health facilities to the rural settings to equitably reach all women.

Married women were 10.74 times more likely to participate in the cervical cancer screening service than unmarried women (AOR = 10.74, 95% CI = 5.02–22.96), according to this study. This finding is in contrast to the finding of studies conducted in different settings, which revealed that married women were less likely to involve in the cervical cancer screening service delivery.

[18, 22]. The difference might be attributed to the geographical and cultural differences in the study areas and the impact of SBCC interventions and cervical cancer screening campaigns on the current study.

This study revealed that birth experience and place of birth were predictors of the uptake of cervical cancer screening service among women aged 15 years old or more. Women who had a birth experience were 8.92 times more likely to receive cervical cancer screening service than women who study participants who have no childbirth experience (AOR = 8.92, 95% CI = 4.28–19.19). This might imply that those women who had a history of birth experience might have better interaction or communication with the health workers during their visit. On the other hand, it might indicate that women had visited health facilities have an opportunity to gain awareness about the cervical cancer screening service uptake, which leads to better utilization of the screening service. Women who gave birth at the health facilities (health center, hospital, health post and private clinics) had higher uptake of cervical cancer screening than those who gave birth at home.

Women who had one or more visit of health facilities per year for any health problems were 3.63 times (AOR = 3.63, 95% CI = 1.86–6.93) more likely to receive cervical cancer screening services from health facilities compared to those women with no history of health facility visits. This finding was supported by the reports of studies conducted at Peru and Kenya [26, 27]. This finding might indicated that while women visited health facilities, they would get adequate information about it or encouraged by health facilities. This study revealed that health workers encouragement as one of the predicting factors identified. Those women encouraged by health workers to use cervical cancer screening services were 3.23 times more likely to utilize the service from health settings than women of reproductive age who had not been visited by community health workers (AOR = 3.23, 95% CI = 1.57–6.63). The finding of this study was supported by the result of a study conducted in Jordan which showed that health workers encouragement improved the cervical cancer screening service uptake [28].

Women of reproductive age who gave birth at the health facilities and had a history of visiting health facilities have better record of cervical cancer screening service utilization. These might indicate that they the health workers provided awareness and encouraged women to uptake the cervical cancer screening service. The study also found that women who had awareness about cervical cancer and cervical cancer screening were 0.37 and 4.52 times more likely to uptake cervical cancer screening services. This finding was supported by the findings of studies conducted in different parts of Ethiopia [17, 18, 20, 22].

Women who were employed in government organizations were 2.61 times more likely to uptake cervical cancer screening service (AOR = 2.61, 95% CI = 1.33–5.15), according to the finding of this study. This report is in agreement with the finding of a study conducted at Jimma [29]. Perceived distance from the screening health facility was one of the perceived predictor for the uptake of cervical cancer screening service reported from the participants of this study. Women aged 15 years or older who have the access to and availability of health facility for receiving cervical cancer screening service were 4.45 times more likely to actively screen for cervical cancer check-ups than study participants who travels longer distance to health facility (AOR = 4.41,95%CI = 2.53–9.41). This finding was supported by the finding of a study conducted in Zimbabwe [30], which showed that long distance from health facilities at which cervical cancer screening was conducted was one of a barrier for poor utilization of the cervical cancer screening service.

Generally, the findings highlights that there is a need to empower women through education and decision making; designing and providing health education program to raise consciousness of women at the health facilities regularly; and, conducting social and behavioral change communication interventions.

## Conclusions

From this study, it was understood that cervical cancer screening practice was low. Women marital status (being married), residence, distance from the screening center, frequency of health facility visits, history of birth experience, husband support, place of birth of the last child, and having good awareness were independent predictors of cervical cancer screening. This study highlights that there is a need to conduct SBCC interventions. Therefore, health care providers should have to conduct SBCC interventions continuously to develop women’s health seeking behavior towards the uptake of cervical cancer screening.

## Data Availability

All the data analyzed in this article are available from the first author and can be obtained upon reasonable request.

## Abbreviations

AOR: Adjusted odds ratio
BCC: Behavior change communication
CI: Confidence interval
COR: Crude odds ratio
HPV: Human papilloma virus
SD: Standard deviation
SPSS: Statistical package for social science
WHO: World Health Organization
